# The cross talk of two family members of β-TrCP in the regulation of cell autophagy and growth

**DOI:** 10.1038/s41418-019-0402-x

**Published:** 2019-08-13

**Authors:** Danrui Cui, Xiaoqing Dai, Jianfeng Shu, Ying Ma, Dongping Wei, Xiufang Xiong, Yongchao Zhao

**Affiliations:** 1 Key Laboratory of Combined Multi-Organ Transplantation, Ministry of Public Health, The First Affiliated Hospital, Zhejiang University School of Medicine, Hangzhou, China; 2 Institute of Translational Medicine, Zhejiang University School of Medicine, Hangzhou, China; 3 Cancer Institute, The Second Affiliated Hospital, Zhejiang University School of Medicine, Hangzhou, China; 4 Department of Oncology, Nanjing First Hospital, Nanjing Medical University, Nanjing, China

**Keywords:** Oncogenes, Macroautophagy, Ubiquitin ligases, Ubiquitin ligases

## Abstract

β-transducin repeat-containing protein (β-TrCP), one of the best-characterized substrate recognition components of the SKP1-CUL1-F-box (SCF) E3 ligase, has two distinct paralogs, β-TrCP1 and β-TrCP2, expressed in mammals. Through governing the ubiquitination and degradation of numerous key regulators, β-TrCP1/2 regulates various cellular physiological and pathological processes. However, whether and how these two proteins cross talk and whether they regulate cell autophagy and proliferation in different manners is completely unknown. Herein, we report that β-TrCP1 and β-TrCP2 are the physiological substrates of SCF E3 ligase and target each other for degradation that is dependent on their β-TrCP degron sequences. Furthermore, glucose deprivation activates AMPK kinase to phosphorylate β-TrCP1 and promotes the subsequent ubiquitination and degradation of β-TrCP1 by β-TrCP2, but does not promote β-TrCP2 degradation by β-TrCP1. Finally, we found that β-TrCP2, not β-TrCP1, preferentially degrades DEPTOR and REDD1, the inhibitors of mTORC1, to activate mTORC1, leading to autophagy inhibition and cell growth. Thus, our study demonstrates that β-TrCP1 and β-TrCP2 mutually target each other for degradation and that β-TrCP2 acts as a dominant paralog in the regulation of cell autophagy and growth, which might be a promising anticancer target.

## Introduction

β-transducin repeat-containing protein (β-TrCP), one of the best characterized mammalian F-box proteins of SCF ligase, plays vital roles in several physiological and pathological processes, including cell cycle progression, signal transduction, and tumorigenesis, by targeting several key regulators for ubiquitination and degradation [1]. Interestingly, β-TrCP, which is highly conserved from *Drosophila* to human, is encoded by a single gene in invertebrates, whereas mammalian genomes contain two distinct paralogs: β-TrCP1 (known as FBXW1) and β-TrCP2 (known as FBXW11). Both paralogs have similar biochemical properties, with an F-box motif at the N-terminus that binds to SKP1 and seven WD40 repeats at the C-terminus, which are involved in the recognition of and interaction with specific substrates. Thus, as a substrate receptor, β-TrCP forms SCF ligase complex, also known as cullin-RING ligase 1 (CRL1), with cullin-1 acting as a molecular scaffold, adaptor protein SKP1, and a RING protein, RBX1, serving as the ligase activity center.

It is well established that only substrates phosphorylated at the binding site can be recognized by F-box proteins [2]. Thus, the substrates of β-TrCP need to be phosphorylated by kinase(s) at a specific binding motif (known as degron motif) and then recognized by β-TrCP. β-TrCP recognizes DSGxxS degron motif or its variants, such as DSG/DDG/EEG/SSG/TSGxxS/E/D motifs, whose serine or threonine residues are phosphorylated by specific kinases [1, 3]. The well-characterized substrates of β-TrCP include several tumor suppressors, such as Bim EL, IκB, PDCD4, and CDC25A, and multiple oncoproteins, such as β-catenin, YAP, and WEE1 [1, 4]. Thus, owing to the diversity of its substrates, β-TrCP has been suggested to possess both tumor-promoting and tumor-suppressing characteristics [5, 6]. Given the importance of β-TrCP in tumorigenesis, its activity or stability should be controlled strictly. However, little is known about the upstream signaling pathways that govern β-TrCP activity and the ubiquitin ligases that are responsible for its ubiquitination and degradation. Thus, elucidating how the activity and stability of β-TrCP are controlled would lead to a better understanding of their growth regulatory role under physiological or stressed conditions (e.g., glucose deprivation).

Here, we reported that β-TrCP1 and β-TrCP2 target each other for degradation. Upon glucose deprivation, β-TrCP1 was rapidly degraded by β-TrCP2 following phosphorylation by AMPK, whereas β-TrCP2 could not be degraded by β-TrCP1. Biologically, we silenced either β-TrCP1 or β-TrCP2 to mimic their degradation and found that β-TrCP2 inhibits autophagy and promotes cell growth via activating mTORC1, whereas β-TrCP1 suppresses cell growth.

## Results

### Both β-TrCP1 and β-TrCP2 are the substrate of CRL1 E3 ligase

**Fig. 1:**
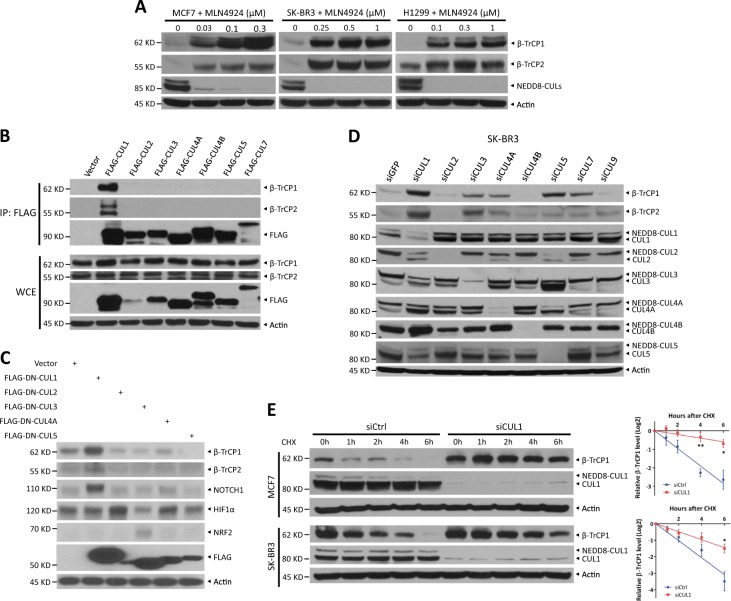
β-TrCP1/2 is a substrate of CRL1 E3 ligase. **a** Cells were treated with various concentrations of MLN4924 for 24 h and harvested for IB with anti-β-TrCP1, β-TrCP2, NEDD8, and Actin Abs. **b** HEK293 cells were transfected with vector and the indicated FLAG-CULs. After 48 h, cells were subjected to immunoprecipitation (IP) with FLAG beads and then IB with anti-β-TrCP1, β-TrCP2, FLAG, and Actin Abs. **c** HEK293 cells were transfected with vector or the indicated FLAG-DN-CULs for 48 h and then harvested for IB with anti-β-TrCP1, β-TrCP2, NOTCH1, HIF1α, NRF2, FLAG, and Actin Abs. **d** SK-BR3 cells were transfected with the indicated siRNA targeting CULs or scrambled control siRNA for 48 h and then subjected to IB with anti-β-TrCP1, β-TrCP2, CUL1, CUL2, CUL3, CUL4A, CUL4B, CUL5, and Actin Abs. **e** MCF7 and SK-BR3 cells were transfected with CUL1 siRNA or scrambled control siRNA for 48 h. Cells were then treated with 100 μg/ml CHX for the indicated time periods and then subjected to IB with anti-β-TrCP1, CUL1, and Actin Abs. Densitometry quantification was performed with ImageJ, and the decay curves are shown (right) (mean ± S.E.M., *n* = 3, *p *<* 0.05, **p < 0.01)

Several F-box proteins, including β-TrCP, are unstable and degraded by proteasomes [7–10]. However, how β-TrCP1/2 protein stability is regulated in vivo remains poorly understood. Consistent with previous studies, we found that MG132 treatment, a proteasome inhibitor, increased endogenous β-TrCP1 levels and significantly extended its protein half-life, indicating that β-TrCP1 is degraded by the ubiquitin-proteasome system (Fig. S1A and S1B). Given that CRLs are the largest ubiquitin ligase family [4, 11], we next determined whether CRLs are the ligase for β-TrCP1 ubiquitination. Indeed, MLN4924, a small molecule inhibitor of NEDD8-activating enzyme that inactivates CRLs by inhibiting cullin neddylation [11], caused β-TrCP1 accumulation in a time- and dose-dependent manner in all tested cells (Figs. 1a and S1C), remarkably extended the protein half-life of β-TrCP1 (Fig. S1D) and decreased β-TrCP1 polyubiquination (Fig. S1E). These results suggest that β-TrCP1 is a physiological substrate of CRLs.

We next determined whether β-TrCP2, like β-TrCP1, is a substrate of CRLs. Interestingly, MLN4924 also remarkably induced β-TrCP2 accumulation in a dose-dependent manner in all tested cells (Fig. 1a). Likewise, MG132 and MLN4924 treatment each extended its protein half-life (Fig. S1F). Furthermore, β-TrCP2 polyubiquitination was significantly increased by MG132 treatment, which was blocked by combinational MLN4924 treatment (Fig. S1G). Taken together, our results indicate that β-TrCP2 is also a substrate of CRLs.

The human genome encodes a total of eight cullins (CUL1, 2, 3, 4 A, 4B, 5, 7, and 9) [4, 12]. In a pull-down assay, we detected the binding of FLAG-tagged CUL1, but no other CULs, to endogenous β-TrCP1 and β-TrCP2 (Fig. 1b). Consistently, only a dominant-negative CUL1 mutant caused the accumulation of endogenous β-TrCP1/2, while dominant-negative mutants of CUL1, CUL2, and CUL3 resulted in increases in NOTCH1, HIF1α, and NRF2, known substrates of CUL1/2/3, respectively, as positive controls (Fig. 1c). Moreover, the levels of β-TrCP1 and β-TrCP2 were increased more dramatically upon knockdown of CUL1 than upon knockdown of each of the other seven cullins (Fig. 1d and S1H). Interestingly, silencing of CUL5 also increased β-TrCP1 levels, which is consistent with a recently published study that SAG-CUL5 negatively regulates β-TrCP1 levels via K11-linkage mediated ubiquitination [13]. In addition, β-TrCP2 levels were also increased upon CUL3 silencing (Fig. 1d). However, CUL3 silencing had no effect on the protein half-life of β-TrCP2 (Fig. S1I) and did not increase, but significantly decreased the mRNA levels of β-TrCP2 (Fig. S1J), suggesting that CUL3 might negatively regulate β-TrCP2 at the translational level. Thus, we next focused on the role of CUL1 in regulating the degradation of β-TrCP1 and β-TrCP2. Indeed, CUL1 silencing significantly extended the protein half-life of β-TrCP1 (Fig. 1e). Together, both β-TrCP1 and β-TrCP2 are degraded by CRL1 or SCF.

### β-TrCP1 is a substrate of SCF^β-TrCP2^ and β-TrCP2 is a substrate of SCF^β-TrCP1^

**Fig. 2:**
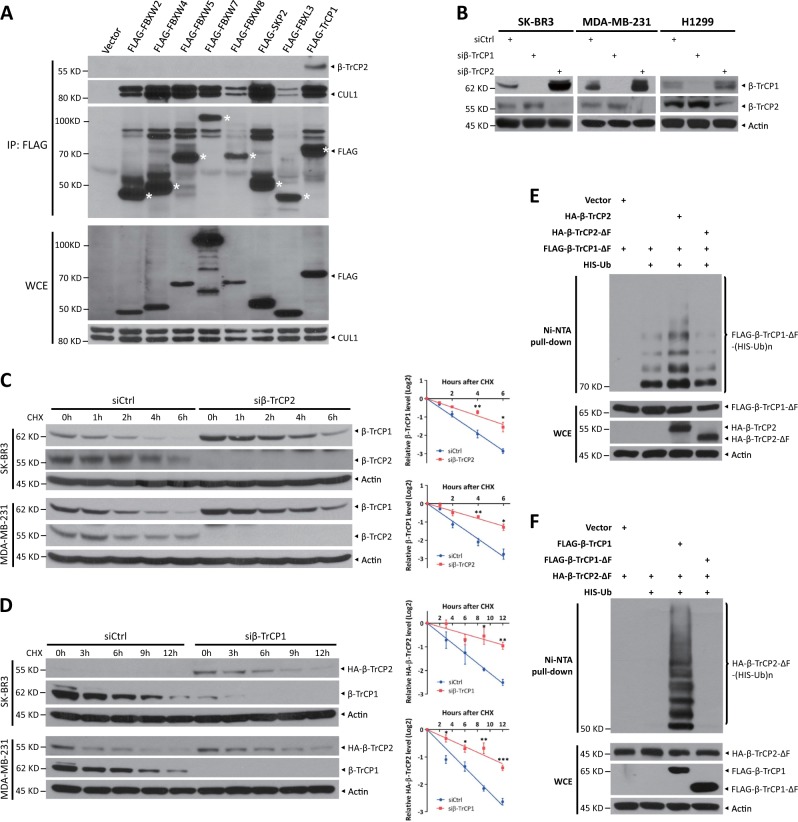
β-TrCP1 is a substrate of SCF^β-TrCP2^ and β-TrCP2 is a substrate of SCF^β-TrCP1^. **a** HEK293 cells were transfected with vector or the indicated FLAG-F-box plasmids for 48 h. Cells were then subjected to IP with FLAG beads and IB with anti-β-TrCP2, CUL1, and FLAG Abs. An asterisk indicates corresponding FLAG-F-box proteins. **b** Cells were transfected with siRNA targeting β-TrCP1 or β-TrCP2 or with scrambled control siRNA for 48 h and then subjected to IB with anti-β-TrCP1, β-TrCP2, and Actin Abs. **c** Cells were transfected with the indicated siRNA for 48 h. Then cells were treated with 100 μg/ml CHX for the indicated time periods and then subjected to IB with anti-β-TrCP1, β-TrCP2, and Actin Abs. **d** Cells with endogenous HA-β-TrCP2 established by CRISPR-Cas9-mediated knock-in were infected with indicated lentiviral shRNA virus for 72 h. Then cells were treated with 100 μg/ml CHX for the indicated time periods and then subjected to IB with anti-HA, β-TrCP1, and Actin Abs. Densitometry quantification was performed with ImageJ, and the decay curves are shown (mean ± S.E.M., *n* = 3, *p *<* 0.05, **p < 0.01, ***p < 0.001) (**c**, **d**, right). **e**, **f** HEK293 cells were transfected with the indicated plasmids for 48 h, lysed under denaturing conditions, and then pulled down by Ni-NTA beads. Pull-downs (top) and whole-cell extracts (bottom) were subjected to IB with anti-FLAG, HA, and Actin Abs. WCE: whole-cell extracts

Next, we explored which F-box protein targets β-TrCP1/2 for ubiquitination. First, of several F-box proteins, only FLAG-tagged β-TrCP1 pulled down endogenous β-TrCP2 (Fig. 2a), suggesting that β-TrCP2 may target β-TrCP1 for ubiquitination, and/or vice versa. Indeed, β-TrCP2 silencing significantly increased β-TrCP1 levels in all tested cells, whereas knockdown of β-TrCP1 moderately increased β-TrCP2 levels (Figs. 2b and S2A). In addition, knockdown of β-TrCP2 or β-TrCP1 had no effect on the mRNA levels of β-TrCP1 or β-TrCP2 (Fig. S2B), respectively, suggesting that the regulation between β-TrCP1 and β-TrCP2 may occur at the posttranslational level. Indeed, the protein half-life of β-TrCP1 was clearly extended by β-TrCP2 knockdown via two different siRNA oligos (Figs. 2c and S2C). On the other hand, the protein half-life of endogenous or exogenous β-TrCP2 was extended upon shRNA-mediated β-TrCP1 knockdown or upon CRISPR-Cas9-mediated β-TrCP1 knockout (Figs. 2d and S2D). Finally, the in vivo ubiquitination assay showed that β-TrCP2, but not β-TrCP2-ΔF, a dominant-negative mutant, promoted β-TrCP1-ΔF polyubiquitination (Fig. 2e), whereas β-TrCP1, but not β-TrCP1-ΔF, boosted β-TrCP2-ΔF polyubiquitination (Fig. 2f). These results suggested that the polyubiquitination of β-TrCP1 and β-TrCP2 was promoted by each other, not by themselves. In addition, CRISPR-Cas9-mediated β-TrCP1 knockout suppressed β-TrCP2 polyubiquitination (Fig. S2E). Taken together, our results suggest that β-TrCP1 is a novel substrate of SCF^β-TrCP2^ and β-TrCP2 is a novel substrate of SCF^β-TrCP1^.

### Ubiquitination of β-TrCP1 and β-TrCP2 is dependent on their degron sequences

**Fig. 3:**
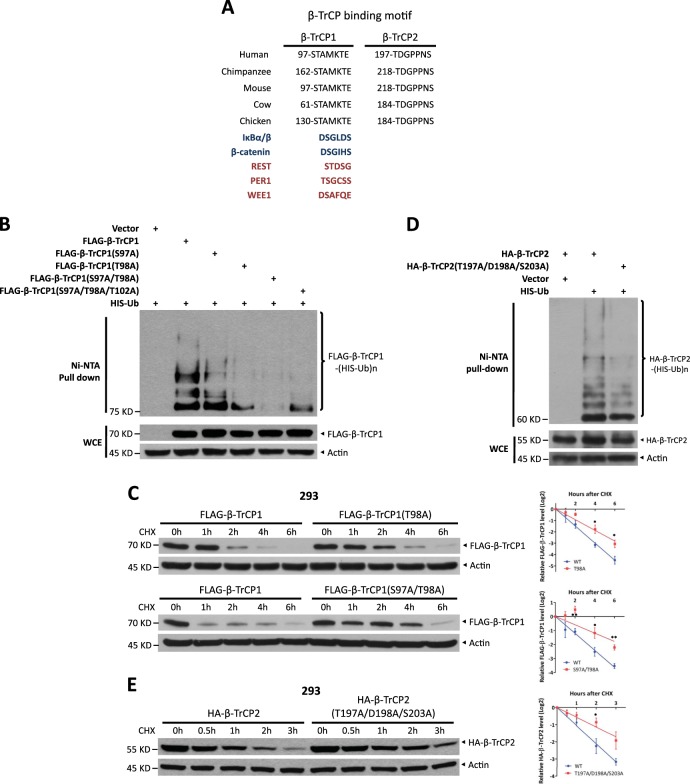
The ubiquitination and stability of β-TrCP1 and β-TrCP2 are dependent on their degron sequences. **a** β-TrCP1 has a putative β-TrCP degron motif (97-STAMKTE-103 in human), and β-TrCP2 also has a putative β-TrCP degron motif (197-TDGPPNS-203 in human), both of which are highly evolutionarily conserved. **b** HEK293 cells were transfected with the indicated plasmids for 48 h, lysed under denaturing conditions and then subjected to pull-down by Ni-NTA beads. Pull-downs (top) and whole-cell extracts (bottom) were subject to IB with anti-FLAG and Actin Abs. **c**, **e** HEK293 cells were transfected with the indicated degron site mutants. After 48 h, cells were treated with 100 μg/ml CHX for the indicated time periods and then subjected to IB with anti-FLAG (**c**), HA (**e**), and Actin Abs. Densitometry quantification was performed with ImageJ, and the decay curves are shown (right) (mean ± S.E.M., *n* = 3, *p *<* 0.05, **p < 0.01). **d** HEK293 cells were transfected with the indicated plasmids for 48 h, lysed under denaturing conditions, and then pulled down by Ni-NTA beads. Pull-downs (top) and whole-cell extracts (bottom) were subjected to IB with anti-HA and Actin Abs

We next examined the sequence of β-TrCP1 and β-TrCP2 for β-TrCP consensus binding motifs and found an evolutionarily conserved atypical degron motif (^97^STAMKTE^103^) for β-TrCP1 and a degron motif variant (^197^TDGPPNS^203^) for β-TrCP2 (Fig. 3a). The polyubiquitination, promoted by endogenous β-TrCP2, of β-TrCP1 mutants in which the key residues within the degron motif were mutated was remarkably lower than that of the wild type (WT) (Fig. 3b). Consequently, the protein half-lives of β-TrCP1 mutants were significantly extended (Fig. 3c and S2F). On the other hand, the polyubiquitination, promoted by endogenous β-TrCP1, of a β-TrCP2 triple mutant was also remarkably lower than that of the WT (Fig. 3d). Furthermore, the protein half-life of this triple mutant was significantly extended (Fig. 3e). However, the interaction between degron motif mutants of β-TrCP1 and WT β-TrCP2, as well as the binding of degron motif mutants of β-TrCP2 with WT β-TrCP1, was not markedly different from WT interactions (Fig. S3A and S3B). These results could be explained by the dual identity of β-TrCP1/2, such as β-TrCP1: (1) as a substrate of SCF^β-TrCP2^, the degron motif mutants of β-TrCP1 could not be recognized by β-TrCP2; however, (2) as a component of SCF^β-TrCP1^, no matter how the degron motif is mutated, β-TrCP1 can interact with β-TrCP2, as the substrate of SCF^β-TrCP1^, through its WD domain. Thus, the degron sequences of β-TrCP1/2 determine their ubiquitination and degradation.

### β-TrCP1 is degraded upon serum starvation or glucose deprivation

**Fig. 4:**
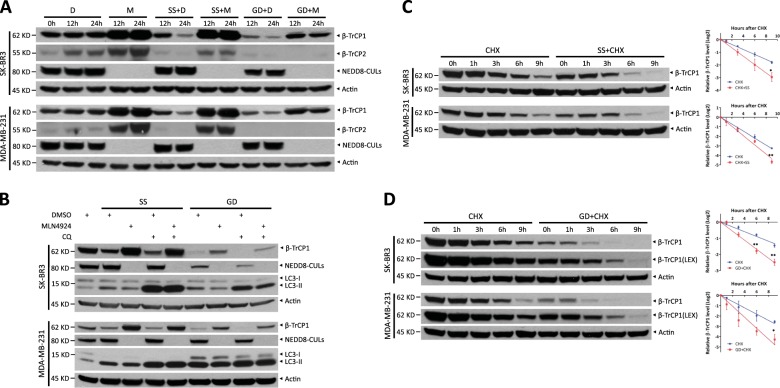
Glucose deprivation and serum starvation each trigger β-TrCP1 degradation. **a** Cells were serum-starved or glucose-deprived for 12 or 24 h in the absence or presence of 1 μM MLN4924, respectively, and then subjected to IB with anti-β-TrCP1, β-TrCP2, NEDD8, and Actin Abs. **b** Cells were serum-starved or glucose-deprived for 24 h, pretreated with 1 μM MLN4924 for 12 h and/or 50 μM CQ for 6 h and then subjected to IB with anti-β-TrCP1, NEDD8, LC3, and Actin Abs. Cells were serum-starved for 24 h (**c**) or glucose-deprived for 12 h (**d**), treated with 100 μg/ml CHX for the indicated time periods and then subjected to IB with anti-β-TrCP1 and Actin Abs. Densitometry quantification was performed with ImageJ, and the decay curves are shown (right) (mean ± S.E.M., *n* = 3, *p *<* 0.05, **p < 0.01). D: DMSO; M: MLN4924; SS: serum starvation; GD: glucose deprivation; LEX: longer exposure

Next, we defined the upstream signaling pathway(s) and potential kinase(s) that govern β-TrCP1/2 stability and function. We exposed cells to several stimuli such as DNA damage, serum stimulation (data not shown), serum starvation, and glucose deprivation, and found that serum starvation and glucose deprivation each decreased β-TrCP1 levels, which was completely abrogated by MLN4924 treatment (Fig. 4a). This result indicates that β-TrCP1 degradation triggered by serum starvation or glucose deprivation depends on the ligase activity of CRLs.

Given that serum starvation or glucose deprivation also induces autophagy, another pivotal degradation system [14], we then determined whether autophagy regulates β-TrCP1 degradation. We found that chloroquine (CQ), an inhibitor of autophagy, did not inhibit β-TrCP1 reduction, whereas MLN4924, an inhibitor of CRLs, blocked the decrease of β-TrCP1 triggered by serum starvation or glucose deprivation (Fig. 4b). This result excluded the possible involvement of autophagy in β-TrCP1 degradation. Furthermore, except for a moderate decrease upon glucose deprivation in SK-BR3 cells, β-TrCP1 mRNA levels were not significantly decreased upon serum starvation or glucose deprivation (Fig. S3C and S3D). Importantly, β-TrCP1 half-life was clearly shortened upon serum starvation or glucose deprivation (Fig. 4c, d). Collectively, our results suggest that serum starvation and glucose deprivation each trigger β-TrCP1 degradation by CRLs, but not by autophagy.

In addition, serum starvation or glucose deprivation also down-regulated β-TrCP2 levels (Fig. 4a). However, MLN4924 treatment only completely abrogated β-TrCP2 reduction by serum starvation, but not by glucose deprivation, suggesting that β-TrCP2 reduction upon glucose deprivation is not mediated by CRLs (Fig. 4a). Furthermore, glucose deprivation did not shorten β-TrCP2 half-life (Fig. S4A), but accelerated β-TrCP1 degradation (Fig. 4d). Taken together, glucose deprivation selectively promoted the degradation of β-TrCP1, but not β-TrCP2, by CRLs. Thus, we next focused on how glucose deprivation triggers β-TrCP1 degradation.

### Activated AMPK triggers β-TrCP1 degradation upon glucose deprivation

**Fig. 5:**
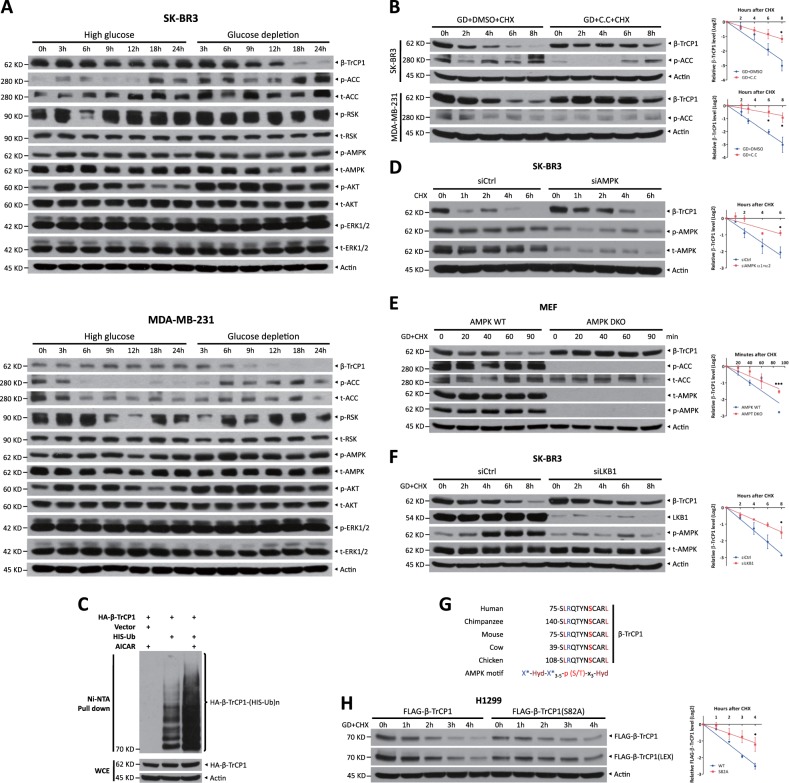
Activated AMPK triggers β-TrCP1 degradation upon glucose deprivation. **a** Cells were cultured in high-glucose or glucose-free medium for the indicated time periods and then harvested for IB with anti-β-TrCP1, p-ACC, t-ACC, p-RSK, t-RSK, p-AMPK, t-AMPK, p-AKT, t-AKT, p-ERK1/2, t-ERK1/2, and Actin Abs. **b** Cells transferred to glucose-free medium were treated with or without 10 μM Compound C for 1 h, treated with 100 μg/ml CHX for the indicated time periods and then subjected to IB with anti-β-TrCP1, p-ACC, and Actin Abs. **c** Cells were pretreated with DMSO or 0.5 mM AICAR for 24 h, lysed under denaturing conditions and then subjected to pull-down by Ni-NTA beads. Pull-downs (top) and whole-cell extracts (bottom) were subject to IB with anti-HA and Actin Abs. SK-BR3 cells were transfected with siRNA targeting AMPKα or scrambled control siRNA for 48 h (**d**); AMPK WT or DKO MEFs were shifted to glucose-free medium for 2 h (**e**), treated with 100 μg/ml CHX for the indicated time periods and then subjected to IB with anti-β-TrCP1, p-AMPK, t-AMPK, p-ACC, t-ACC, and Actin Abs. **f** SK-BR3 cells were transfected with siRNA targeting LKB1 or scrambled control siRNA. After 48 h, cells were shifted to glucose-free medium for 4 h, treated with 100 μg/ml CHX for the indicated time periods and then subjected to IB with anti-β-TrCP1, LKB1, p-AMPK, t-AMPK, and Actin Abs. **g** Human β-TrCP1 has a putative AMPK motif (75-SLRQTYNSCARL-86) that is highly evolutionarily conserved. Hyd: bulky hydrophobic residues (like L, I, M, F, and V); X^*^: one of these sites is a basic residue. **h** H1299 cells were transfected with FLAG-β-TrCP1 WT or S82A mutant plasmid. After 48 h, cells were shifted to glucose-free medium for 4 h, treated with 100 μg/ml CHX for the indicated time periods and then subjected to IB with anti-FLAG and Actin. Densitometry quantification was performed with ImageJ, and the decay curves are shown (mean ± S.E.M., *n* = 3, *p *<* 0.05, **p < 0.01, ***p < 0.001) (**b**, **d–f**, **h**, right). GD: glucose deprivation; C.C: Compound C; LEX: longer exposure. “t-” denotes antibodies that recognize total protein rather than a specifically modified version

To define which kinase(s) mediate β-TrCP1 degradation in response to glucose deprivation, we first examined the correlation between β-TrCP1 levels and the activity of several kinases upon glucose deprivation and found an inverse correlation between β-TrCP1 levels and AMPK activity, as reflected by the phosphorylation of the AMPK substrate acetyl-CoA carboxylase (ACC) (Fig. 5a). Furthermore, β-TrCP1 degradation induced by glucose deprivation was abrogated by Compound C, an AMPK inhibitor, but not by other tested selective inhibitors, including LY294002 (an AKT inhibitor), BI-D1870 (an inhibitor of RSK1/2), GSK3i-IX (an inhibitor of GSK3), D4476 (a CK1 inhibitor), and CX-4945 (a CK2 inhibitor) (Fig. 5b and S4B–S4F). In contrast, selectively activating AMPK by AICAR treatment significantly promoted β-TrCP1 polyubiquitination (Fig. 5c) and shortened its protein half-life (Fig. S5). More specifically, simultaneous knockdown of AMPKα1 and AMPKα2, the catalytic subunits of AMPK complex, extended β-TrCP1 half-life (Fig. 5d). In addition, β-TrCP1 degradation in *AMPKα1/α2* double-knockout (DKO) mouse embryonic fibroblasts (MEFs) was significantly inhibited, compared with that in paired *AMPK* WT MEFs (Fig. 5e). Furthermore, silencing of LKB1, a well-known upstream activator of AMPK [15], significantly suppressed AMPK activity (reflected by a reduced phosphorylation of AMPK), resulting in the significant extension of β-TrCP1 half-life (Fig. 5f).

To define the precise AMPK phosphorylation site, we examined the β-TrCP1 protein sequence and found that an evolutionarily conserved sequence (^75^SLRQTYN**S**CARL^86^) near the β-TrCP degron motif (^97^STAMKTE^103^) matched the predicted AMPK consensus motif [16, 17] (Fig. 5g). Thus, we proposed that AMPK, acting as a priming kinase, directly phosphorylates β-TrCP1 on Ser82 and facilitates the phosphorylation of the β-TrCP degron motif by other kinase(s). In support of this hypothesis, we mutated Ser82 to Ala and found that β-TrCP1 protein half-life was remarkably extended upon glucose deprivation (Fig. 5h), indicating that mutating Ser82 to Ala may abolish the subsequent phosphorylation of the β-TrCP1 degron motif (S97/T98/T102), leading to β-TrCP1 stabilization. The regulation pattern for phosphorylation of the degron motif by a priming kinase has also been proven in the degradation of β-catenin and YAP, two well-characterized substrates of SCF^β-TrCP^ [18, 19].

Taken together, our results support the notion that upon glucose deprivation, activated AMPK, as a priming kinase, phosphorylates β-TrCP1 at Ser82 to facilitate the phosphorylation of the degron motif for β-TrCP1 degradation.

### β-TrCP1 and β-TrCP2 regulate autophagy and growth differently via modulating the mTOR pathway

**Fig. 6:**
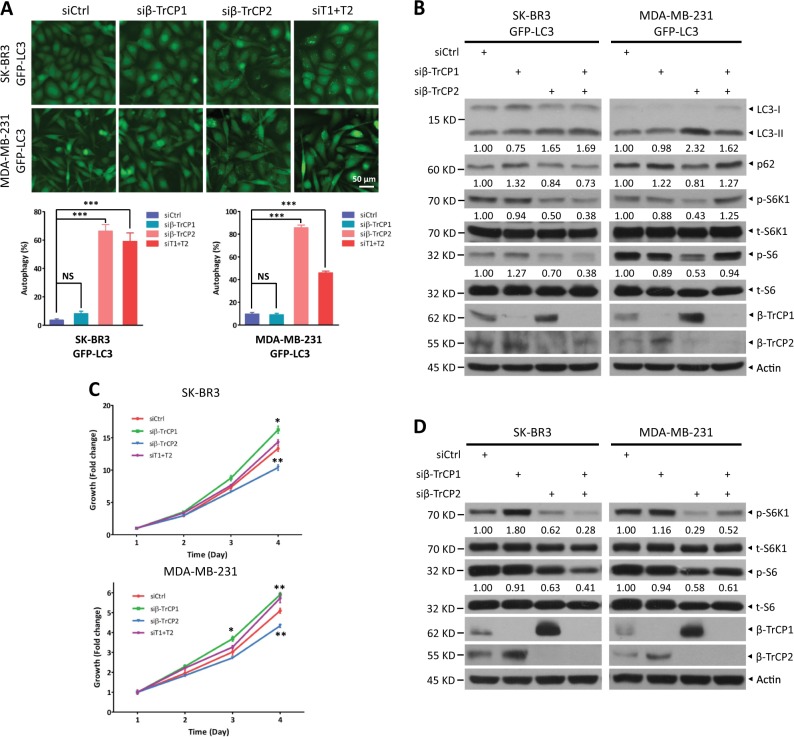
β-TrCP1 and β-TrCP2 regulate autophagy and growth in different manners via modulating mTORC1. SK-BR3 and MDA-MB-231 cells stably expressing GFP-LC3 were transfected with siRNA targeting β-TrCP1, β-TrCP2, or β-TrCP1 + 2 or with a scrambled control siRNA for 48 h. Cells were then photographed under a fluorescence microscope (**a**, top) and subjected to IB (**b**) with anti-LC3, p62, p-S6K1, t-S6K1, p-S6, t-S6, β-TrCP1, β-TrCP2, and Actin Abs. Cells with punctate structures of GFP-LC3 were counted, and their number was expressed as percentage of autophagy (**a**, bottom) (mean ± S.E.M., *n* = 5, ***p *<* 0.001, NS, not significant, compared with cells transfected with scramble control siRNA oligos). SK-BR3 and MDA-MB-231 cells were transfected with the indicated siRNA for 48 h and then subjected to an ATPlite cell growth assay (mean ± S.E.M., *n* = 3, *p *<* 0.05, **p < 0.01, compared with cells transfected with scramble control siRNA oligos) (**c**) or IB with anti-p-S6K1, t-S6K1, p-S6, t-S6, β-TrCP1, β-TrCP2, and Actin Abs (**d**). The band density was quantified and expressed as fold change compared with the corresponding control by setting the control value as 1 (**b**, **d**)

Given the critical role of β-TrCP1/2 in regulation of various cellular physiological and pathological processes, a fundamental question is whether the degradation of β-TrCP1/2 affects cell function. To investigate biological significance of their degradation by each other, we silenced either β-TrCP1 or β-TrCP2 separately to mimic their individual degradation and then analyzed the phenotypes of the cells, including cell autophagy, senescence, migration, and growth. Given that β-TrCP1 and β-TrCP2 target each other for degradation, the loss of function from β-TrCP2 silencing may be compromised by β-TrCP1 accumulation, thus resulting in no cellular phenotype upon knockdown of β-TrCP1 or β-TrCP2, if the two β-TrCP paralogs are functionally redundant. Interestingly, siRNA silencing of β-TrCP2 but not β-TrCP1 dramatically induced autophagy by two different targeting sequences, as reflected by LC3-positive puncta formation, a hallmark of autophagy (Figs. 6a and S6A). Consistently, the conversion of LC3-I to LC3-II and p62 degradation, two markers to measure autophagy flux [20], was induced by β-TrCP2 silencing (Fig. 6b). These results suggest that the functions of β-TrCP2 and β-TrCP1 are not redundant. Furthermore, autophagy induction by β-TrCP2 silencing was higher than that by silencing both β-TrCP1/2, indicating that β-TrCP2 accumulation by β-TrCP1 silencing may partially compromise β-TrCP2 decrease by siRNA knockdown. Consistently, β-TrCP2 silencing suppressed cell growth, β-TrCP1 silencing promoted cell growth inversely and silencing of both β-TrCP2 and β-TrCP1 had minor, if any, effect on cell growth (Fig. 6c). Mechanistically, the activity of mTORC1, a key governor coordinating autophagy and cell growth [21], was inhibited upon β-TrCP2 silencing, as reflected by reduced phosphorylation of S6K1 and S6 (Fig. 6b, d). Conversely, β-TrCP1 silencing activated mTORC1 slightly, if at all, as reflected by increased S6K1 phosphorylation (Fig. 6d). Interestingly, although mTORC1 activity was clearly suppressed upon silencing of both β-TrCP2 and β-TrCP1 (Fig. 6d), cell growth was not inhibited (Fig. 6c), which may be due to the complexity of SCF^β-TrCP^ substrates. In addition, silencing of β-TrCP2, but not β-TrCP1, significantly induced senescence and inhibited cell migration, as reflected by β-gal positive staining and wound-healing assays, respectively (Fig. S6B and S6C). Collectively, our results support the notion that of the two paralogs, β-TrCP2 plays a dominant role in regulation of cell autophagy, growth, senescence, and migration.

### β-TrCP2 regulates mTORC1 activity by selectively targeting DEPTOR and REDD1 for degradation

**Fig. 7:**
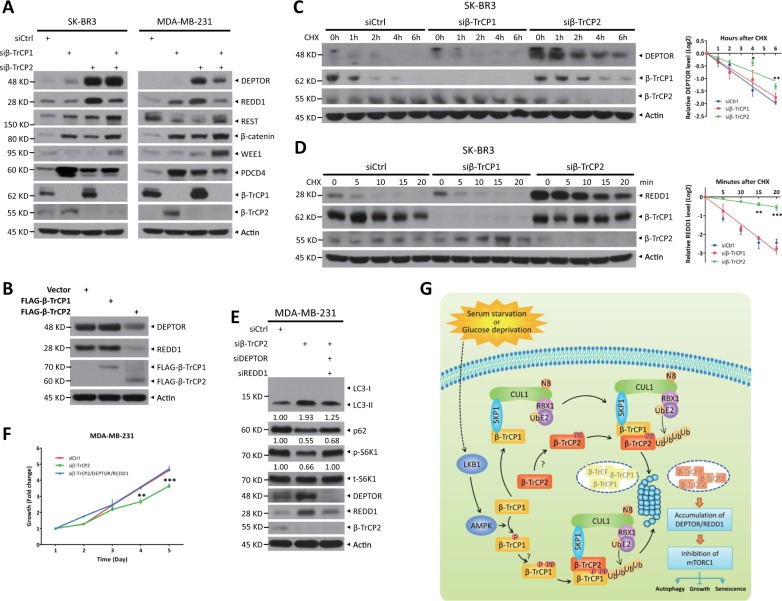
DEPTOR and REDD1, inhibitors of mTORC1, are degraded preferentially by β-TrCP2. **a** Cells were transfected with indicated siRNA for 48 h and then harvested for IB with anti-DEPTOR, REDD1, REST, β-catenin, WEE1, PDCD4, β-TrCP1, β-TrCP2, and Actin Abs. **b** HEK293 cells were transfected with the indicated plasmids for 48 h and then subjected to IB with anti-DEPTOR, REDD1, FLAG, and Actin Abs. **c**, **d** SK-BR3 cells were transfected with indicated siRNA for 48 h. In addition, cells were then treated with 100 μg/ml CHX for the indicated time periods and then subjected to IB with anti-DEPTOR, REDD1, β-TrCP1, β-TrCP2, and Actin Abs. Densitometry quantification was performed with ImageJ, and the decay curves are shown (right) (mean ± S.E.M., *n* = 3, *p *<* 0.05, **p < 0.01, ***p < 0.001, compared with cells transfected with scramble control siRNA oligos). MDA-MB-231 cells were transfected with the indicated siRNA for 48 h and then subjected to IB with anti-LC3, p62, p-S6K1, t-S6K1, DEPTOR, REDD1, β-TrCP2, and Actin Abs (**e**), or ATPlite cell growth assay (mean ± S.E.M., *n* = 3, **p < 0.01, ***p < 0.001, compared with cells transfected with scramble control siRNA oligos) (**f**). The band density was quantified and expressed as fold change compared with the corresponding control by setting the control value as 1 (**e**). **g** A model for β-TrCP1/2 degradation and the distinct roles of β-TrCP1/2 in the regulation of mTORC1

Given that β-TrCP1/2 is a substrate receptor of SCF ligase complex, their functional difference may be explained through their selectivity for substrates. We next examined the abundance of various well-established substrates upon β-TrCP1/2 depletion. DEPTOR [22–24] and REDD1 [25, 26], two known mTORC1 inhibitors and β-TrCP substrates, were accumulated to much greater levels in β-TrCP2-depleted cells than in β-TrCP1-depleted cells (Fig. 7a). The levels of other substrates, including REST, β-catenin, WEE1, and PDCD4, change, if at all, minimally upon the depletion of β-TrCP2 or β-TrCP1 (Fig. 7a). Furthermore, overexpression of β-TrCP2 rather than β-TrCP1 significantly reduced the levels of DEPTOR and REDD1 (Fig. 7b). And their protein half-lives were clearly extended upon β-TrCP2 silencing but not upon β-TrCP1 silencing (Fig. 7c, d). Finally, simultaneous silencing of DEPTOR and REDD1 in β-TrCP2-depleted cells restored mTORC1 activity, as demonstrated by the recovery of S6K1 phosphorylation, and partially suppressed β-TrCP2 knockdown induced autophagy, as reflected by partial inhibition of the conversion of LC3-I to LC3-II and p62 degradation (Fig. 7e), leading to the abrogation of growth inhibition by β-TrCP2 silencing (Fig. 7f). Taken together, these results demonstrate that DEPTOR and REDD1 are degraded preferentially by β-TrCP2 and required for autophagy induction and growth inhibition by β-TrCP2 silencing.

## Discussion

In this study, we characterized β-TrCP1 and β-TrCP2, two paralogs in mammals, as the physiological substrates of SCF ligase and showed that they target each other for ubiquitination. It is worth noting that β-TrCP1 and β-TrCP2 interacted with themselves (Fig. S7A and S7B), indicating the possibility of self-degradation. Similarly, FBXW7, another well-characterized F-box protein, suffers from self-ubiquitination-mediated degradation by the Pin1 prolyl isomerase [8]. Emerging evidence suggests that homodimerization of the F-box protein enhances its ligase activity toward downstream substrates [27, 28]. β-TrCP1/2 contains a D-domain, which is known as a dimerization domain. A previous study showed that a β-TrCP homodimer, not a heterodimer, selectively promoted IκBα degradation upon TNFα treatment [29]. Moreover, our finding indicates that β-TrCP1 promoted ubiquitination of β-TrCP2-ΔF and β-TrCP2 boosted ubiquitination of β-TrCP1-ΔF (Fig. 2e, f), indicating that β-TrCP1 and β-TrCP2 are actually the substrates of another β-TrCP paralog. Thus, our findings of negative cross talk between these two β-TrCP paralogs added another layer of complexity in self-regulation of the F-box proteins. It is conceivable that β-TrCP1/2 homodimerization promotes their E3 activity against the substrates and that heterodimerization mediates their own degradation.

Both β-TrCP paralogs with similar biochemical properties are thought to be functionally redundant due to their lack of selectivity in the recognition of substrates. Moreover, *β-TrCP1*^−/−^ male mice showed only moderate impairment in spermatogenesis and *β-TrCP1*^−/−^ female mice were normal, indicating the compensation of β-TrCP2 for β-TrCP1 depletion [30, 31]. However, in our study, β-TrCP1 and β-TrCP2 have different effects on regulation of cell processes: β-TrCP2 inhibits autophagy and senescence and promotes cell proliferation and migration, whereas β-TrCP1 suppresses cell growth, and have minor, if any, effects on cell autophagy, senescence, and migration (Figs. 6 and S6), suggesting that β-TrCP1/2 are functionally nonredundant. Mechanistically, β-TrCP2 regulates autophagy and proliferation by activating mTORC1, preferentially degrading DEPTOR and REDD1 (Fig. 7). In contrast, β-TrCP1 significantly suppresses cell growth, likely by targeting oncoproteins for degradation, such as β-catenin and YAP [1, 4]. Indeed, β-catenin induction upon β-TrCP1 silencing was higher than that upon β-TrCP2 silencing (Figs. 7a and S7C). Future study is required to determine the causal role of β-catenin in growth induction by β-TrCP1 silencing. Thus, our study showed that β-TrCP1 and β-TrCP2 have selectivity for certain unique substrates. Likewise, in response to virus infection, β-TrCP2 has also been shown to play a nonredundant role [32]. Consistent with our findings that β-TrCP2 silencing inhibits cell growth (Fig. 6c), the proliferation of *β-TrCP2*^Δ/–^ MEFs is markedly lower than that of WT MEFs; this decrease can be rescued by β-TrCP2 overexpression, but not by β-TrCP1 overexpression [33]. Collectively, β-TrCP1 and β-TrCP2 play nonredundant roles in substrate recognition as well as in the regulation of cell processes.

Why is β-TrCP2 more effective than β-TrCP1 in the degradation of unique substrates such as DEPTOR and REDD1? It is possibly attributed to different cellular localization of both paralogs. Several studies showed that β-TrCP1 and β-TrCP2 are mainly expressed in the nucleus and the cytoplasm, respectively [34, 35]. Consistently, DEPTOR and REDD1, mainly localized in the cytoplasm [36, 37], are degraded preferentially by β-TrCP2. However, using nuclear and cytoplasm fractionation assay, we found that although β-TrCP1 was also localized in the cytoplasm, DEPTOR and REDD1 accumulated in cytoplasm when β-TrCP2, not β-TrCP1, was silenced (Fig. S7C), indicating that cytosolic DEPTOR and REDD1 are indeed preferentially degraded by β-TrCP2. Thus, the selectivity of substrate recognition by β-TrCP1/2 might be involved in some unknown regulatory mechanisms, which warrants further investigations.

The diversity of β-TrCP substrates, including both oncoproteins and tumor suppressors, makes it difficult to determine whether β-TrCP is an oncoprotein or a tumor suppressor [1, 6, 38]. Our results showed that β-TrCP2 silencing induces autophagy and senescence and suppresses cell growth and migration, indicating the oncogenic properties of β-TrCP2. Consistently, β-TrCP2 depletion inhibits the proliferation of MEFs [33]. Conversely, β-TrCP2 overexpression accelerates lymphocytic leukemia cell proliferation in vitro and tumorigenesis in vivo [39]. Therefore, targeted inactivation of β-TrCP2 possessing oncogenic properties might be a promising anticancer therapy strategy.

Although mammalian genomes contain two different β-TrCP1/2 genes, located in chromosomes 10 and 5, respectively, there is only a single β-TrCP gene in invertebrates, which is β-TrCP2 [35, 38]. Moreover, compared with β-TrCP1, β-TrCP2 is more conserved in evolution, proving its importance in the maintenance of basic physiological functions among species. Furthermore, the studies using knockout mice certified the vital role of β-TrCP2 in development. *β-TrCP1*^−/−^ mice displayed only minor defects in spermatogenesis [30, 31], whereas *β-TrCP2*^−/−^ mice died before embryonic day 10.5 [33]. These findings and our study support the notion that β-TrCP2 is the dominant paralog of β-TrCP in governing cell processes.

In summary, our study reveals a previously unknown interplay and difference between two β-TrCP paralogs in mammals. F-box proteins β-TrCP1 and β-TrCP2, components of SCF ligase, target each other for degradation. In response to glucose deprivation or serum starvation, AMPK is activated and phosphorylates β-TrCP1 for subsequent SCF^β-TrCP2^-mediated ubiquitination and degradation. On the other hand, SCF^β-TrCP1^ promotes β-TrCP2 ubiquitination and degradation mediated by unknown kinase(s). More importantly, the degradation of β-TrCP1, mimicked by siRNA silencing, has different effects, compared with the degradation of β-TrCP2 (mimicked in the same manner) on cell autophagy and growth. β-TrCP2 silencing induces autophagy and blocks cell growth, whereas β-TrCP1 silencing promotes cell growth. Mechanistically, β-TrCP2, not β-TrCP1, governs the activity of mTORC1, a central regulator of autophagy and growth, by preferentially degrading DEPTOR and REDD1, two well-known substrates of SCF^β-TrCP^ and inhibitors of mTORC1. Thus, β-TrCP2 acts as a dominant paralog with oncogenic properties in the regulation of cell autophagy and growth (Fig. 7g).

## Methods

### Cell lines and drug treatment

HEK293, MCF7, SK-BR3, MDA-MB-231, A549, H1299, HeLa, and MEF cells were maintained in Dulbecco’s modified Eagle’s medium supplemented with 10% (v/v) fetal bovine serum (FBS). HCT116 cells were maintained in McCoy’s 5 A medium containing 10% FBS. SK-BR3 cells stably expressing HA-β-TrCP2 were established by geneticin (10131–027; GIBCO) selection after transfection with an expression vector encoding HA-β-TrCP2. The pool of HEK293 cells with β-TrCP1 knockout by CRISPR-Cas9 was established by puromycin (Selleck) selection after transfection with sequence-verified CRISPR plasmid. Target sequence of sgβ-TrCP1 is 5′-TTG CTA AAC ATA CTG TTT CT-3′. SK-BR3 and MDA-MB-231 cells with endogenous HA-β-TrCP2 were established by knocking in a 3×HA tag at the N-terminus of endogenous β-TrCP2 using CRISPR-Cas9 technology, which were confirmed by sequencing and immunoblotting (IB). AMPK WT and DKO MEFs were kindly provided by Dr Zongping Xia. SK-BR3 and MDA-MB-231 cells stably expressing GFP-LC3 used in this study were described previously [40]. The following chemicals were obtained from commercial sources: MLN4924 (B1036; Apexbio), MG132 (10012628; Cayman), CHX (C7698; Sigma), Compound C (P5499; Sigma), AICAR (A9978; Sigma), LY294002 (S1105; Selleck), BI-D1870 (S2843; Selleck), GSK3i-IX (HY-10580; MCE), D4476 (S7642; Selleck), and CX-4945 (S2248; Selleck).

### IB and immunoprecipitation (IP)

For direct IB analysis, whole cells were lysed in lysis buffer (50 mM Tris pH 7.5, 0.15 M NaCl, 1% NP-40, 0.1% SDS, 0.5% sodium deoxycholate, 50 mM NaF, 1 mM EDTA, 1 mM DTT, 1 mM Na_3_VO_4_) with protease inhibitors and phosphatase inhibitors. After incubation on ice for 30 min, the supernatant was harvested by spinning at 14,000 rpm for 25 min at 4 °C, followed by the measurement of protein concentration using the BCA protein assay kit (23225; Thermo) and Western blotting as previously described [22]. The following antibodies (Abs) were used: β-TrCP1 (4394; Cell Signaling Technology; 1:1000), NEDD8 (ab81264; Abcam; 1:4000), HA (11867423001; Roche; 1:2000), FLAG (F1804; Sigma; 1:2000), Actin (A5441; Sigma; 1:10,000), NOTCH1 (4147; Cell Signaling Technology; 1:1000), HIF1α (sc-10790; Santa Cruz; 1:500), CUL1 (sc-11384; Santa Cruz; 1:1000), CUL2 (610778; BD Biosciences; 1:200), CUL3 (2759; Cell Signaling Technology; 1:1000), CUL4A (2699; Cell Signaling Technology; 1:1000), CUL4B (12916–1-AP; Proteintech; 1:1000), CUL5 (sc-13014; Santa Cruz; 1:500), p-ACC (3661; Cell Signaling Technology; 1:1000), t-ACC (3676; Cell Signaling Technology; 1:1000), p-RSK (9341; Cell Signaling Technology; 1:1000), t-RSK (9355; Cell Signaling Technology; 1:1000), p-AMPK (2535; Cell Signaling Technology; 1:1000), t-AMPK (5831; Cell Signaling Technology; 1:1000), p-AKT (4060; Cell Signaling Technology; 1:2000), t-AKT (4691; Cell Signaling Technology; 1:2000), p-ERK1/2 (9101; Cell Signaling Technology; 1:1000), t-ERK1/2 (4696; Cell Signaling Technology; 1:1000), LKB1 (3047; Cell Signaling Technology; 1:1000), p62 (PM045; MBL; 1:10,000), LC3 (2775; Cell Signaling Technology; 1:1000), p-S6K1 (9234; Cell Signaling Technology; 1:1000), t-S6K1 (sc-230; Santa Cruz; 1:1000), p-S6 (4858; Cell Signaling Technology; 1:1000), t-S6 (2217; Cell Signaling Technology; 1:1000), DEPTOR (11816; Cell Signaling Technology; 1:1000), REDD1 (10638-1-AP; Proteintech; 1:1000), REST (22242-1-AP; Proteintech; 1:1000), β-catenin (9582; Cell Signaling Technology; 1:1000), WEE1 (4936; Cell Signaling Technology; 1:1000), PDCD4 (9535; Cell Signaling Technology; 1:1000), PARP (9542; Cell Signaling Technology; 1:1000), caspase-3 (9665; Cell Signaling Technology; 1:1000), c-MYC (5605; Cell Signaling Technology; 1:1000), p-GSK3α/β (9331; Cell Signaling Technology; 1:1000), t-GSK3α/β (5676; Cell Signaling Technology; 1:1000), and ubiquitin (3933; Cell Signaling Technology; 1:1000). A human anti-β-TrCP2 polyclonal antibody targeting amino acids 16–243 of β-TrCP2 (Gene ID: 23291) was generated and affinity-purified by Youke Biotech. “p-” and “t-” denote Abs that recognize phosphorylated and total protein, respectively.

To immunoprecipitate exogenously expressed FLAG-tagged or HA-tagged proteins, cells were lysed in lysis buffer (50 mM Tris pH 7.5, 0.15 M NaCl, 1% NP-40, 0.1% SDS, 0.5% sodium deoxycholate, 50 mM NaF, 1 mM EDTA, 1 mM DTT, 1 mM Na_3_VO_4_) with protease inhibitors for 30 min on ice. The whole-cell lysates were harvested after spinning at 14,000 rpm for 30 min at 4 °C, and then the supernatants were then incubated with bead-conjugated FLAG (A2220; Sigma) or bead-conjugated HA (A2095; Sigma) in a rotating incubator for 3 h at 4 °C. The immunoprecipitates were washed with lysis buffer and subjected to Western blotting.

For subcellular fractionation, cells were fractionated into nuclear and cytoplasm fractions using the Cell Fractionation Kit (9038; Cell Signaling Technology). Proteins were separated and immunoblotted as described previously [22].

All the IB and IP experiments were independently repeated at least twice and the representative results are shown. And the protein decay curves are shown as mean ± S.E.M. of three independent experiments.

### In vivo ubiquitination

HEK293 cells were transfected with the indicated plasmids with PolyJet (SL100688; SignaGen Laboratories) for 48 h and pretreated with 1 μM MLN4924, 20 μM MG132, or 0.5 mM AICAR for the indicated time periods as described. Then, the cells were lysed in 6 M guanidine denaturing solution and incubated with Ni-NTA agarose (1018244; Qiagen), as described previously [22].

### Quantitative RT-PCR

Total RNA was isolated from cells using TRIzol reagent (15596018; Invitrogen) and then transcribed into cDNA using the PrimeScript RT reagent kit (RR037A; Takara). Quantitative real-time PCR (qRT-PCR) was performed using SYBR Premix Ex Taq (RR420A; Takara) on an Applied Biosystems Viia7 real-time PCR system. The primers used for qRT-PCR were as follows: 5′-CCT CAT ACT TGC CCA GGA CC-3′ and 5′-AGG TGC AGA GGT GAA AGG AGG-3′ for β-TrCP1; 5′-CAC TTT CAC CCG TTT CAG GG-3′ and 5′-ATT CTG GGT AGG TGT TTG GCA-3′ for β-TrCP2; 5′-AGG GCA TCC TGG GCT ACA C-3′ and 5′-GCC AAA TTC GTT GTC ATA CCA G-3′ for GAPDH.

### siRNA and lentiviral shRNA silencing

Cells were transfected with the following siRNA oligos by Lipofectamine 3000 (L3000-015; Invitrogen). siCtrl: 5′-ATT GTA TGC GAT CGC AGA C-3′; siCUL1: 5′-GGT CGC TTC ATA AAC AAC A-3′; siCUL2: 5′-GAG CTA GCA TTG GAT ATG TGG-3′; siCUL3: 5′-GAA GGA ATG TTT AGG GAT A-3′; siCUL4A: 5′-GAA CTT CCG AGA CAG ACC T-3′; siCUL4B: 5′-AAG CCT AAA TTA CCA GAA A-3′; siCUL5: 5′-GTC TCA CTT CCT ACT GAA CTG-3′; siCUL7: 5′-AAC TGC CAT GTC TAC AAG AAG-3′; siCUL9: 5′-GCT GAG AGA CAC GTT GTT TAG-3′; siβ-TrCP1: 5′-GCG TTG TAT TCG ATT TGA TAA-3′; siβ-TrCP1-B: 5′-GCT GAA CTT GTG TGC AAG GAA-3′; siβ-TrCP2: 5′-TCG TAC TCT CAA TGG GCA CAA-3′; siβ-TrCP2-B: 5′-GTC CAG TAA ATT GCT AAG TAA-3′; siβ-TrCP2-C: 5′-GGT TGT TAG TGG ATC ATC A-3′; siβ-TrCP1 + 2: 5′-AAG TGG AAT TTG TGG AAC ATC-3′; siAMPKα1: 5′-CAA AGT CGA CCA AAT GAT A-3′; siAMPKα2: 5′-GCA TAC CAT CTT CGT GTA AGA-3′; siLKB1: 5′-CAT CTA CAC TCA GGA CTT CAC-3′; siDEPTOR: 5′-GCC ATG ACA ATC GGA AAT CTA-3′; and siREDD1: 5′-GTG GAG ACT AGA GGC AGG AGC-3′. Short hairpins targeting β-TrCP1 (targeting sequence: 5′-GCG TTG TAT TCG ATT TGA TAA-3′) and CUL3 (targeting sequence: 5′-GAA GGA ATG TTT AGG GAT A-3′) were cloned into pLKO.1-puro vector. Lentiviral shRNA virus packaging and subsequent infection of cells were implemented according to the protocol described previously [41].

### ATPlite assay

SK-BR3 and MDA-MB-231 cells were transfected with siRNA targeting β-TrCP1, β-TrCP2, or β-TrCP1 + β-TrCP2 or with scrambled control siRNA for 48 h, and cells were then seeded in 96-well plates (2 × 10^3^ per well). ATPlite assays (6016731; Perkin-Elmer) for cell growth were performed at different time points according to manufacturer’s instructions. The data were obtained from three independent experiments that were each run in triplicate.

### SA-β-gal staining

SK-BR3 cells were transfected with the indicated siRNA oligos for 48 h, transferred to serum-free medium and incubated for 48 h. The expression of senescence-associated β-gal was determined by SA-β-gal staining [33]. For each sample, SA-β-gal-positive and total cell numbers were counted from 5 random microscopic fields.

### Wound-healing assay

SK-BR3 cells were transfected with the indicated siRNA for 48 h and transferred to serum-free medium for 12 h. Then, monolayer cells were scratched with a pipette tip, and the migration into the gap was imaged at the indicated time points using phase-contrast microscopy. For each sample and time point, relative wound confluence was counted from five random microscopic fields.

### Statistical analysis

Two-tailed Student’s *t*-tests for statistical analyses were performed with the data from three independent biological replicates using the Statistical Program for Social Sciences software 20.0 (SPSS, Chicago, IL, USA) to compare parameters between groups. *P* values <0.05 were considered to indicate statistical significance.

## Supplementary information

Supplemental information

## Data Availability

The authors declare that all data supporting the findings of this study are available with the article or from the corresponding author upon reasonable request.
