# Supplementary material for: The cross talk of two family members of β-TrCP in the regulation of cell autophagy and growth
Source: Cell Death Differ. 2019 Aug 13;27(3):1119–33. doi: 10.1038/s41418-019-0402-x (PMC7206145; doi:10.1038/s41418-019-0402-x)
Supplement: Supplementary file 1 — Supplemental information [file 41418_2019_402_MOESM1_ESM.pdf]

Supplemental information

Supplemental figure legends

Figure S1. β-TrCP1/2 is a substrate of CRL1 E3 ligase.

(A) MG132 treatment causes β-TrCP1 accumulation in a time-dependent manner: MCF7 and SK-BR3 cells were treated with 20 μM MG132 for the indicated time periods and then harvested for IB with anti-β-TrCP1 and Actin Abs. (B) MG132 treatment extends the protein half-life of β-TrCP1: Cells were treated with 100 μg/ml CHX with or without 20 μM MG132 for the indicated time periods and then subjected to IB with anti-β-TrCP1 and Actin Abs. Densitometry quantification was performed with ImageJ, and the decay curves are shown (right). (C) MLN4924 treatment causes β-TrCP1 accumulation in a time-dependent manner: Cells were treated with or without 1 μM MLN4924 for the indicated time periods and then subjected to IB with anti-β-TrCP1, CUL1, and Actin Abs. (D) Cells were treated with 100 μg/ml CHX with or without 1 μM MLN4924 for the indicated time periods and then subjected to IB with anti-β-TrCP1, CUL1, and Actin Abs. Densitometry quantification was performed with ImageJ, and the decay curves are shown (right). (E and G) Reduced ubiquitination of β-TrCP1 (E) and β-TrCP2 (G) by MLN4924 treatment. HEK293 cells were transfected with the indicated plasmids for 48 hr. After being pretreated with 20 μM MG132 for 5 hr and/or 1 μM MLN4924 for 24 hr, the cells were lysed under denaturing conditions and then subjected to Ni-NTA-bead pull-down. Pull-downs (top) and whole-cell extracts (bottom) were subjected to IB with anti-FLAG, HA and CUL1 Abs. (F) HEK293 cells were transfected with HA-β-TrCP2 for 48 hr. Cells were then treated with 100 μg/ml CHX with 20 μM MG132 or 1 μM MLN4924 for the indicated time periods and then subjected to IB with anti-HA, CUL1 and Actin Abs. (H) CUL1 silencing increases β-TrCP1 levels: HEK293T and A549 cells were transfected with the indicated siRNA for 48 hr and then subjected to IB with anti-β-TrCP1, CUL1, CUL2, CUL3, CUL4A, CUL4B, CUL5, and Actin Abs. (I) CUL3 silencing has no effect on the protein half-life of β-TrCP2: HEK293 cells were infected with indicated lentiviral shRNA virus for 48 hr and then transfected

with HA-β-TrCP2 for 48 hr, treated with 100 μg/ml CHX for the indicated times and then subjected to IB with anti-HA, CUL3, and Actin Abs. Densitometry quantification was performed with ImageJ, and the decay curves are shown (right) (mean ±S.E.M., n=2). (J) CUL3 silencing decreases the mRNA levels of β-TrCP2: SK-BR3 cells were transfected with CUL3 siRNA or scrambled control siRNA for 72 hr and then subjected to qRT-PCR (mean ±S.E.M., n=3, $^{***}p < 0.001$). WCE: whole-cell extracts.

Figure S2. β-TrCP1 is a substrate of $SCF^{\beta\text{-TrCP2}}$, and β-TrCP2 is a substrate of $SCF^{\beta\text{-TrCP1}}$.

(A) β-TrCP1 silencing increases β-TrCP2 levels: SK-BR3 cells stably expressing HA-β-TrCP2 were transfected with the indicated siRNA oligos for 48 hr and then harvested for IB with anti-HA, β-TrCP1, and Actin Abs. (B) β-TrCP2 silencing does not increase the levels of β-TrCP1 mRNA, and β-TrCP1 silencing does not increase the levels of β-TrCP2 mRNA: cells were transfected with the indicated siRNA oligos for 48 hr and then subjected to qRT-PCR (mean ±S.E.M., n=3, $^{*}p < 0.05$, NS, not significant). (C) β-TrCP2 silencing extends β-TrCP1 protein half-life: cells were transfected with the indicated siRNA oligos for 48 h and then treated with 100 μg/ml CHX for the indicated time periods and subjected to IB with anti-β-TrCP1 and Actin Abs. (D) β-TrCP1 depletion extends the β-TrCP2 half-life: Upon CRISPR/Cas9-based knockout of β-TrCP1 or introduction of the CRISPR/Cas9 control, 293 cells were transfected with HA-β-TrCP2 for 48 hr, treated with 100 μg/ml CHX for the indicated times and then subjected to IB with anti-HA, β-TrCP1, and Actin Abs. (E) β-TrCP1 depletion decreases β-TrCP2 polyubiquitination: the pool of β-TrCP1 knockout or CRISPR-Cas9 control 293 cells were transfected with the indicated plasmids for 48 hr, lysed under denaturing conditions and then subjected to pull-down by Ni-NTA beads. Pull-downs (top) and whole-cell extracts (bottom) were subjected to IB with anti-FLAG, β-TrCP1, and Actin Abs. (F) The β-TrCP1 mutant has a longer protein half-life: H1299 cells were transfected with the indicated degron site mutant. After 48 hr, cells were treated with 100 μg/ml CHX for the indicated time periods and then subjected to IB with anti-FLAG and Actin Abs. Densitometry quantification was performed with ImageJ, and the decay curves are shown (C, D and F, right).

Figure S3. The interaction between two β-TrCP mutants and the effect of serum starvation or glucose deprivation on the mRNA levels of β-TrCP1.

(A and B) HEK293 cells were transfected with the indicated plasmids for 48 hr and then subjected to IP with HA beads (A) or FLAG beads (B) and IB with anti-β-TrCP1, CUL1, HA, FLAG and Actin Abs. (C and D) SK-BR3 (C) and MDA-MB-231 (D) cells were subjected to serum starvation or glucose deprivation for 12 or 24 hr, followed by qRT-PCR analysis (mean±S.E.M., n=3, **p* < 0.05, ***p* < 0.01, NS, not significant).

Figure S4. Glucose deprivation selectively promotes β-TrCP1 degradation, which cannot be blocked by selective inhibition of several kinases.

(A) Glucose deprivation did not shorten the protein half-life of β-TrCP2: cells with endogenous HA-β-TrCP2 established by CRISPR-Cas9-mediated knock-in were glucose-deprived for 12 hr, treated with 100 μg/ml CHX for the indicated time periods and then subjected to IB with anti-HA, p-ACC, t-ACC and Actin Abs. Densitometry quantification was performed with ImageJ, and the decay curves are shown (right). (B-F) SK-BR3 and MDA-MB-231 cells were subjected to glucose deprivation in the absence or presence of 25 nM LY294002 (B), 10 μM BI-D1870 (C), 10 μM GSK3i-IX (D), 50 μM D 4476 (E), or 10 μM CX-4945 (F) for 4 hr (B and C) or 6 hr (D-F). Cells were then treated with 100 μg/ml CHX for the indicated time periods and subjected to IB with anti-β-TrCP1, p-AKT, t-AKT, p-GSK3α/β, t-GSK3α/β, c-MYC, and Actin Abs.

Figure S5. Activated AMPK triggers β-TrCP1 degradation.

Activation of AMPK by AICAR treatment shortens the β-TrCP1 half-life: cells were pretreated with DMSO or 0.5 mM AICAR for 12 hr, treated with 100 μg/ml CHX for the indicated time periods and then subjected to IB with anti-β-TrCP1, p-ACC, and Actin Abs.

Figure S6. β-TrCP1 and β-TrCP2 regulate autophagy and growth in different manners.

(A) β-TrCP2 silencing induces autophagy: SK-BR3 and MDA-MB-231 cells stably expressing GFP-LC3 were transfected with the indicated siRNA for 48 hr. Cells were

photographed under a fluorescence microscope (left). Cells with punctate structures of GFP-LC3 were counted, and their number was expressed as the percentage of autophagy (right) (mean ±S.E.M., n=5, ***p* < 0.01, ****p* < 0.001, NS, not significant, compared to cells transfected with scramble control siRNA). (B) β-TrCP2 silencing induces senescence upon serum starvation: SK-BR3 cells were transfected with the indicated siRNA for 48 hr and then shifted to serum-free medium for 48 hr. Representative phase-contrast images of the cells lacking serum for 0 hr (left, top) or 48 hr (left, middle) are shown. In addition, senescence cells were detected with SA-β-gal staining (left, bottom). Cells with β-gal positive staining were counted, and their number was expressed as the percentage of senescence (right) (mean ±S.E.M., n=5, ***p* < 0.01, ****p* < 0.001, NS, not significant, compared to cells transfected with scramble control siRNA). (C) β-TrCP2 silencing suppresses cell migration: SK-BR3 cells were transfected with the indicated siRNA for 48 hr, shifted to serum-free medium for 12 hr and then subjected to a wound-healing assay. The images were photographed under phase-contrast microscopy (left). Relative wound confluence was measured and expressed as migration index (right) (mean ±S.E.M., n=5, **p* < 0.05, ***p* < 0.01, ****p* < 0.001, NS, not significant, compared to cells transfected with scramble control siRNA).

Figure S7. β-TrCP1 and β-TrCP2 form homodimers and DEPTOR and REDD1 accumulated in cytoplasm upon β-TrCP2 silencing.

(A and B) β-TrCP1 and β-TrCP2 interact with themselves: HEK293 cells were transfected with the indicated plasmids for 48 hr. Cells were then subjected to IP with FLAG beads (A) or HA beads (B) and then IB with anti-HA, FLAG, and Actin Abs. (C) Silencing of β-TrCP2, but not β-TrCP1, accumulates DEPTOR and REDD1 in cytoplasm: SK-BR3 cells transfected with the indicated siRNA were subjected to nuclear and cytoplasm fractionation, followed by IB with anti-DEPTOR, REDD1, β-catenin, β-TrCP1, β-TrCP2, PARP, and caspase-3 Abs. PARP and caspase-3 served as markers of the nuclear and cytoplasmic fractions, respectively.

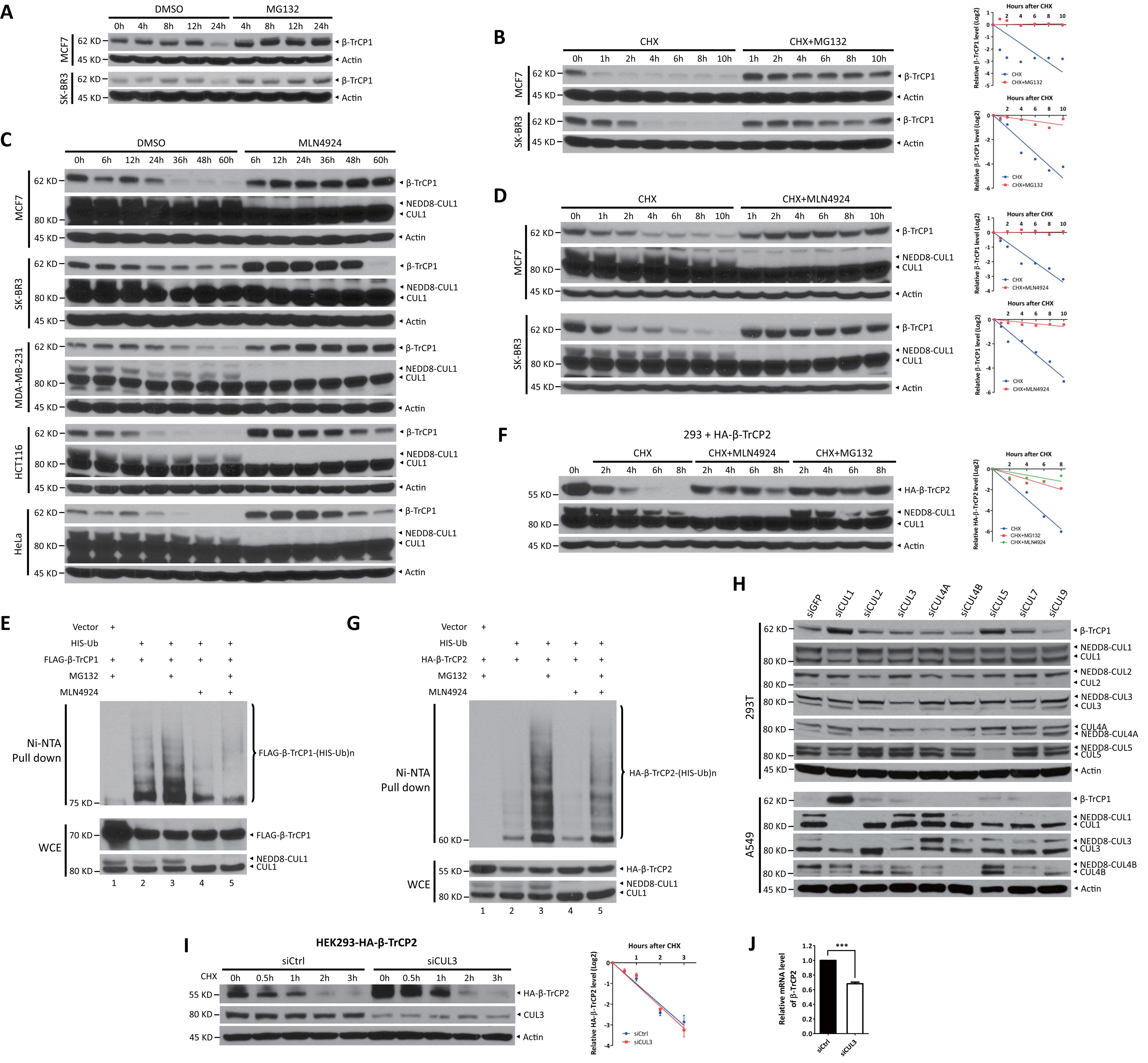
A
DMSO
MG132
0h 4h 8h 12h 24h 4h 8h 12h 24h
MCF7
SK-BR3
62 KD
45 KD
β-TrCP1
Actin
B
CHX
CHX+MG132
0h 1h 2h 4h 6h 8h 10h 1h 2h 4h 6h 8h 10h
Hours after CHX
Relative β-TrCP1 level (Log2)
C
DMSO
MLN4924
0h 6h 12h 24h 36h 48h 60h 6h 12h 24h 36h 48h 60h
MDA-MB-231
HCT116
HeLa
80 KD
NEDD8-CUL1
CUL1
D
CHX+MLN4924
F
293 + HA-β-TrCP2
55 KD
HA-β-TrCP2
Relative HA-β-TrCP2 level (Log2)
E
Vector
HIS-Ub
FLAG-β-TrCP1
MG132
MLN4924
Ni-NTA
Pull down
FLAG-β-TrCP1-(HIS-Ub)n
75 KD
70 KD
WCE
FLAG-β-TrCP1
1 2 3 4 5
G
HA-β-TrCP2
HA-β-TrCP2-(HIS-Ub)n
60 KD
H
siGFP siCUL1 siCUL2 siCUL3 siCUL4A siCUL4B siCUL5 siCUL7 siCUL9
293T
A549
NEDD8-CUL2
CUL2
NEDD8-CUL3
CUL3
CUL4A
NEDD8-CUL4A
NEDD8-CUL5
CUL5
NEDD8-CUL4B
CUL4B
I
HEK293-HA-β-TrCP2
siCtrl
siCUL3
0h 0.5h 1h 2h 3h
Relative HA-β-TrCP2 level (Log2)
J
Relative mRNA level of β-TrCP2
***
siCtrl
siCUL3

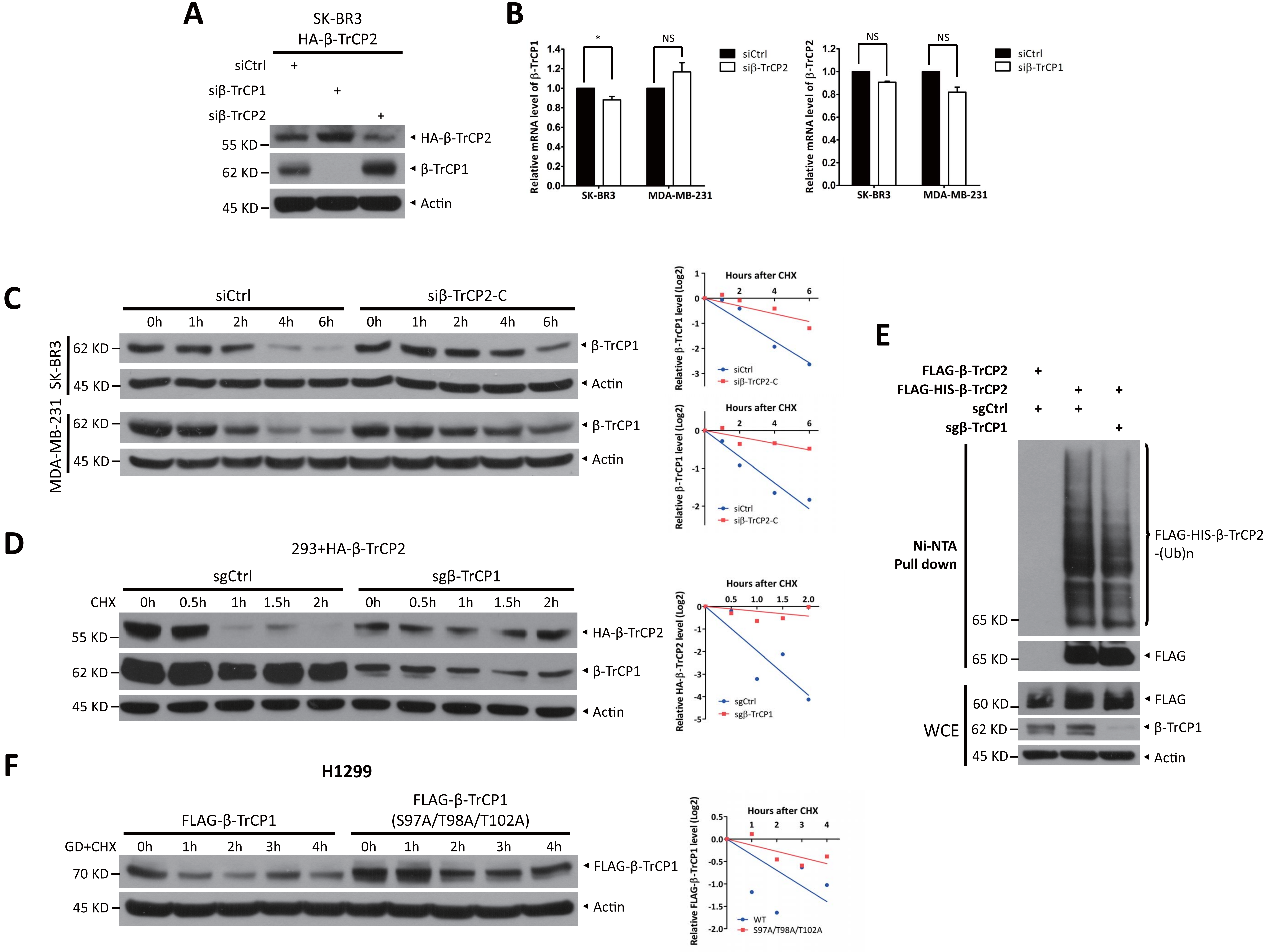
A
SK-BR3
HA-β-TrCP2
siCtrl
siβ-TrCP1
siβ-TrCP2
55 KD
62 KD
45 KD
HA-β-TrCP2
β-TrCP1
Actin
B
Relative mRNA level of β-TrCP1
Relative mRNA level of β-TrCP2
SK-BR3
MDA-MB-231
*
NS
siCtrl
siβ-TrCP2
siβ-TrCP1
C
siCtrl
siβ-TrCP2-C
0h
1h
2h
4h
6h
SK-BR3
MDA-MB-231
62 KD
45 KD
β-TrCP1
Actin
Hours after CHX
Relative β-TrCP1 level (Log2)
D
293+HA-β-TrCP2
sgCtrl
sgβ-TrCP1
CHX
0h
0.5h
1h
1.5h
2h
55 KD
62 KD
45 KD
HA-β-TrCP2
β-TrCP1
Actin
Relative HA-β-TrCP2 level (Log2)
E
FLAG-β-TrCP2
FLAG-HIS-β-TrCP2
sgCtrl
sgβ-TrCP1
Ni-NTA
Pull down
65 KD
FLAG-HIS-β-TrCP2
-(Ub)n
FLAG
WCE
60 KD
62 KD
45 KD
F
H1299
FLAG-β-TrCP1
FLAG-β-TrCP1
(S97A/T98A/T102A)
GD+CHX
0h
1h
2h
3h
4h
70 KD
45 KD
FLAG-β-TrCP1
Actin
Relative FLAG-β-TrCP1 level (Log2)
WT
S97A/T98A/T102A

Cui et al. Figure S3

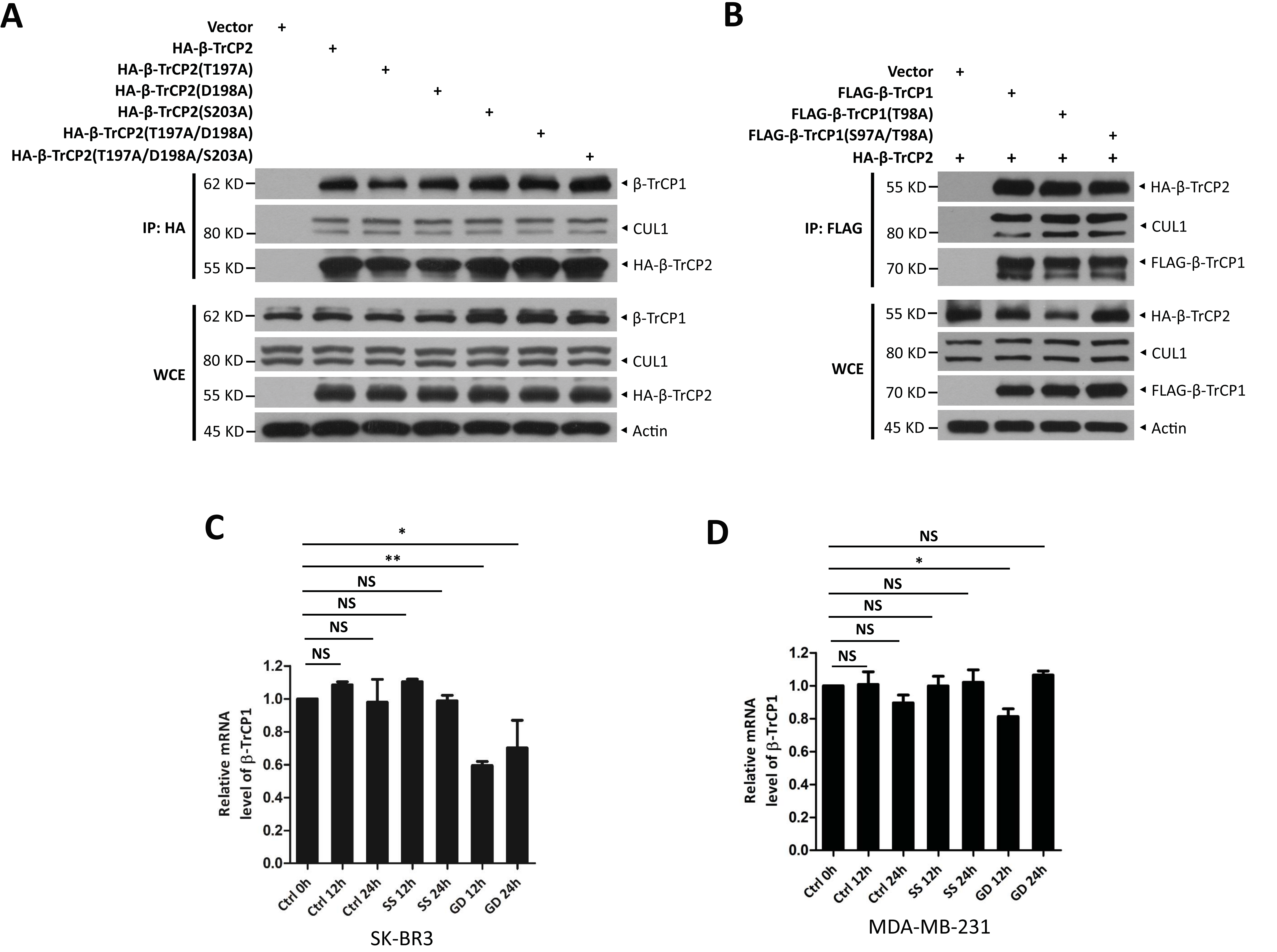

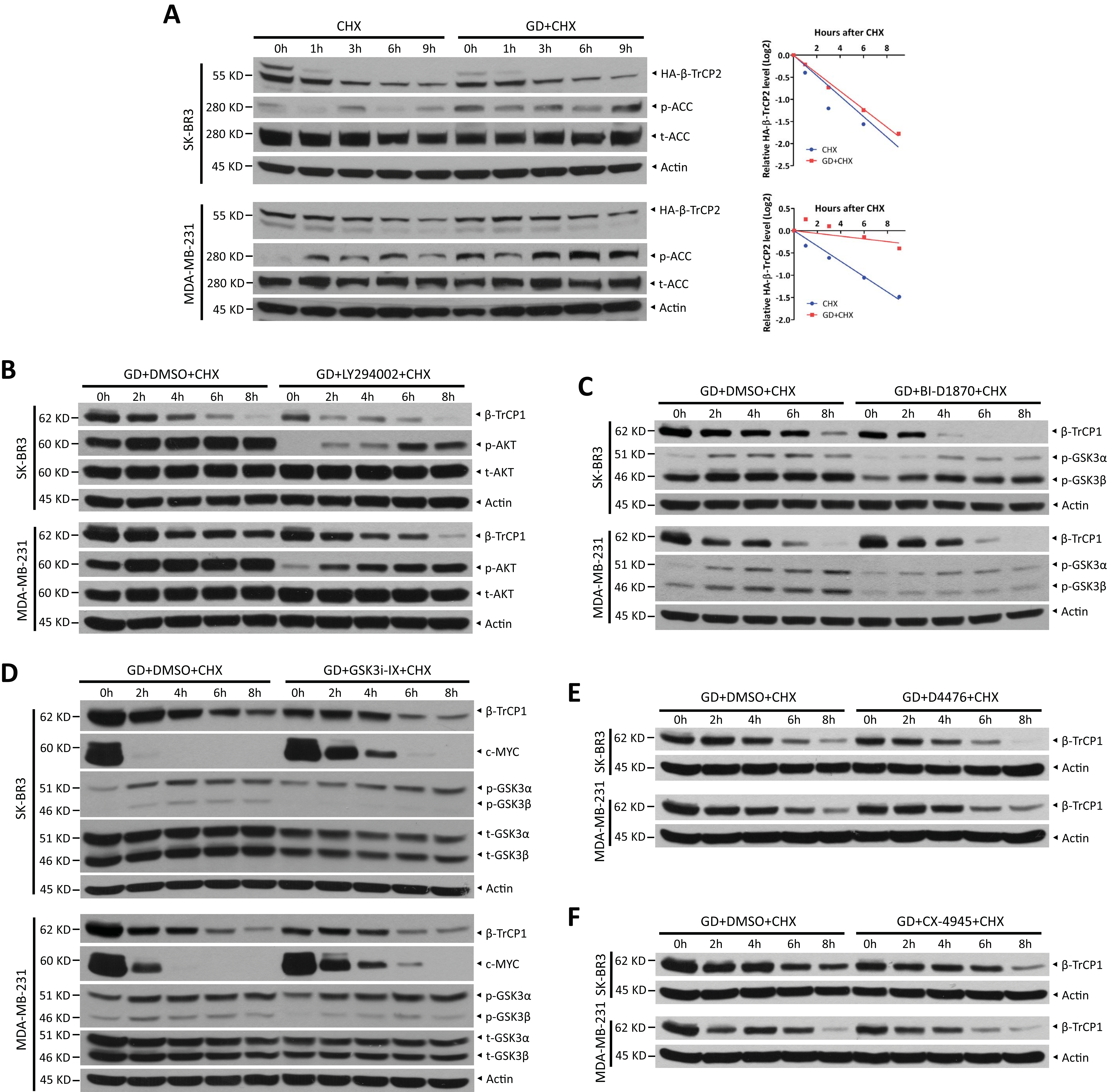
A
CHX
GD+CHX
0h 1h 3h 6h 9h 0h 1h 3h 6h 9h
SK-BR3
55 KD
280 KD
280 KD
45 KD
HA-β-TrCP2
p-ACC
t-ACC
Actin
MDA-MB-231
55 KD
280 KD
280 KD
45 KD
HA-β-TrCP2
p-ACC
t-ACC
Actin
Hours after CHX
2 4 6 8
0.0 -0.5 -1.0 -1.5 -2.0 -2.5
Relative HA-β-TrCP2 level (Log2)
CHX
GD+CHX
Hours after CHX
2 4 6 8
0.5 0.0 -0.5 -1.0 -1.5 -2.0
Relative HA-β-TrCP2 level (Log2)
CHX
GD+CHX
B
GD+DMSO+CHX
GD+LY294002+CHX
0h 2h 4h 6h 8h 0h 2h 4h 6h 8h
SK-BR3
62 KD
60 KD
60 KD
45 KD
β-TrCP1
p-AKT
t-AKT
Actin
MDA-MB-231
62 KD
60 KD
60 KD
45 KD
β-TrCP1
p-AKT
t-AKT
Actin
C
GD+DMSO+CHX
GD+BI-D1870+CHX
0h 2h 4h 6h 8h 0h 2h 4h 6h 8h
SK-BR3
62 KD
51 KD
46 KD
45 KD
β-TrCP1
p-GSK3α
p-GSK3β
Actin
MDA-MB-231
62 KD
51 KD
46 KD
45 KD
β-TrCP1
p-GSK3α
p-GSK3β
Actin
D
GD+DMSO+CHX
GD+GSK3i-IX+CHX
0h 2h 4h 6h 8h 0h 2h 4h 6h 8h
SK-BR3
62 KD
60 KD
51 KD
46 KD
51 KD
46 KD
45 KD
β-TrCP1
c-MYC
p-GSK3α
p-GSK3β
t-GSK3α
t-GSK3β
Actin
MDA-MB-231
62 KD
60 KD
51 KD
46 KD
51 KD
46 KD
45 KD
β-TrCP1
c-MYC
p-GSK3α
p-GSK3β
t-GSK3α
t-GSK3β
Actin
E
GD+DMSO+CHX
GD+D4476+CHX
0h 2h 4h 6h 8h 0h 2h 4h 6h 8h
SK-BR3
62 KD
45 KD
β-TrCP1
Actin
MDA-MB-231
62 KD
45 KD
β-TrCP1
Actin
F
GD+DMSO+CHX
GD+CX-4945+CHX
0h 2h 4h 6h 8h 0h 2h 4h 6h 8h
SK-BR3
62 KD
45 KD
β-TrCP1
Actin
MDA-MB-231
62 KD
45 KD
β-TrCP1
Actin

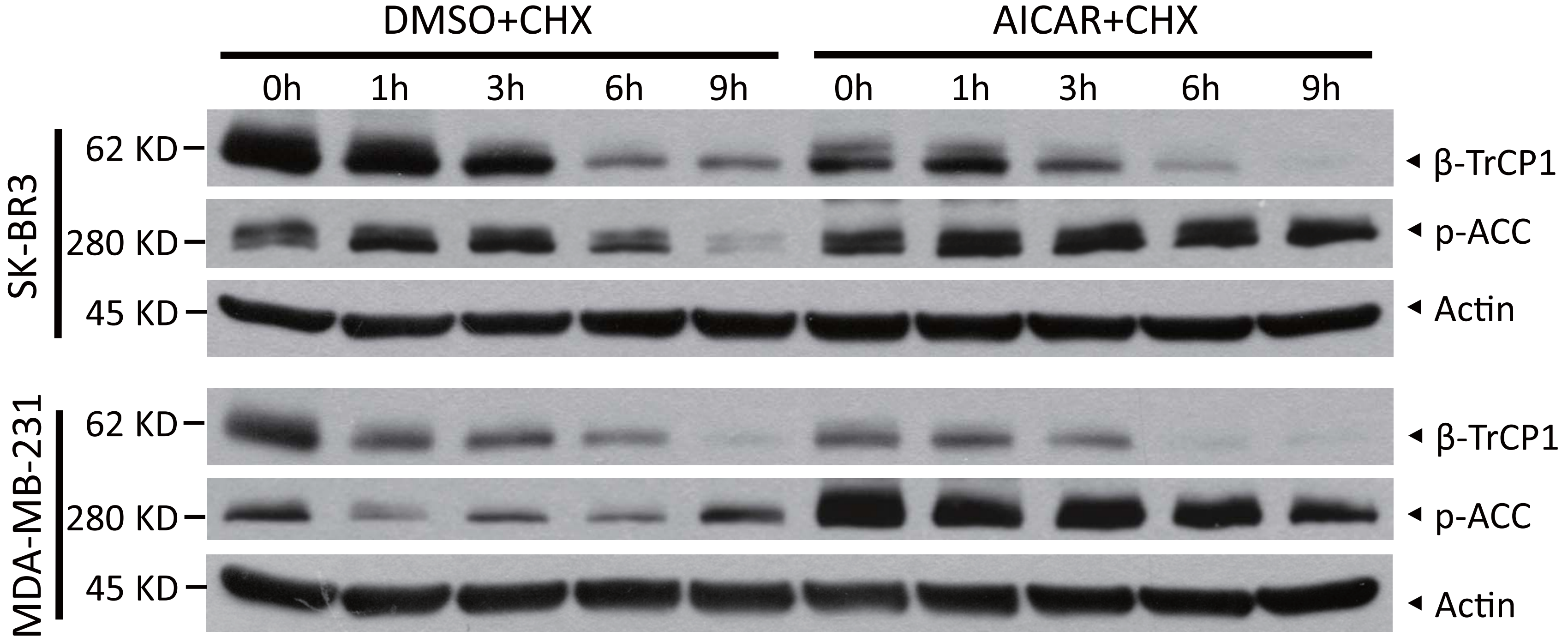
DMSO+CHX
AICAR+CHX
0h
1h
3h
6h
9h
0h
1h
3h
6h
9h
SK-BR3
62 KD
280 KD
45 KD
β-TrCP1
p-ACC
Actin
MDA-MB-231
62 KD
280 KD
45 KD
β-TrCP1
p-ACC
Actin

Cui et al. Figure S6

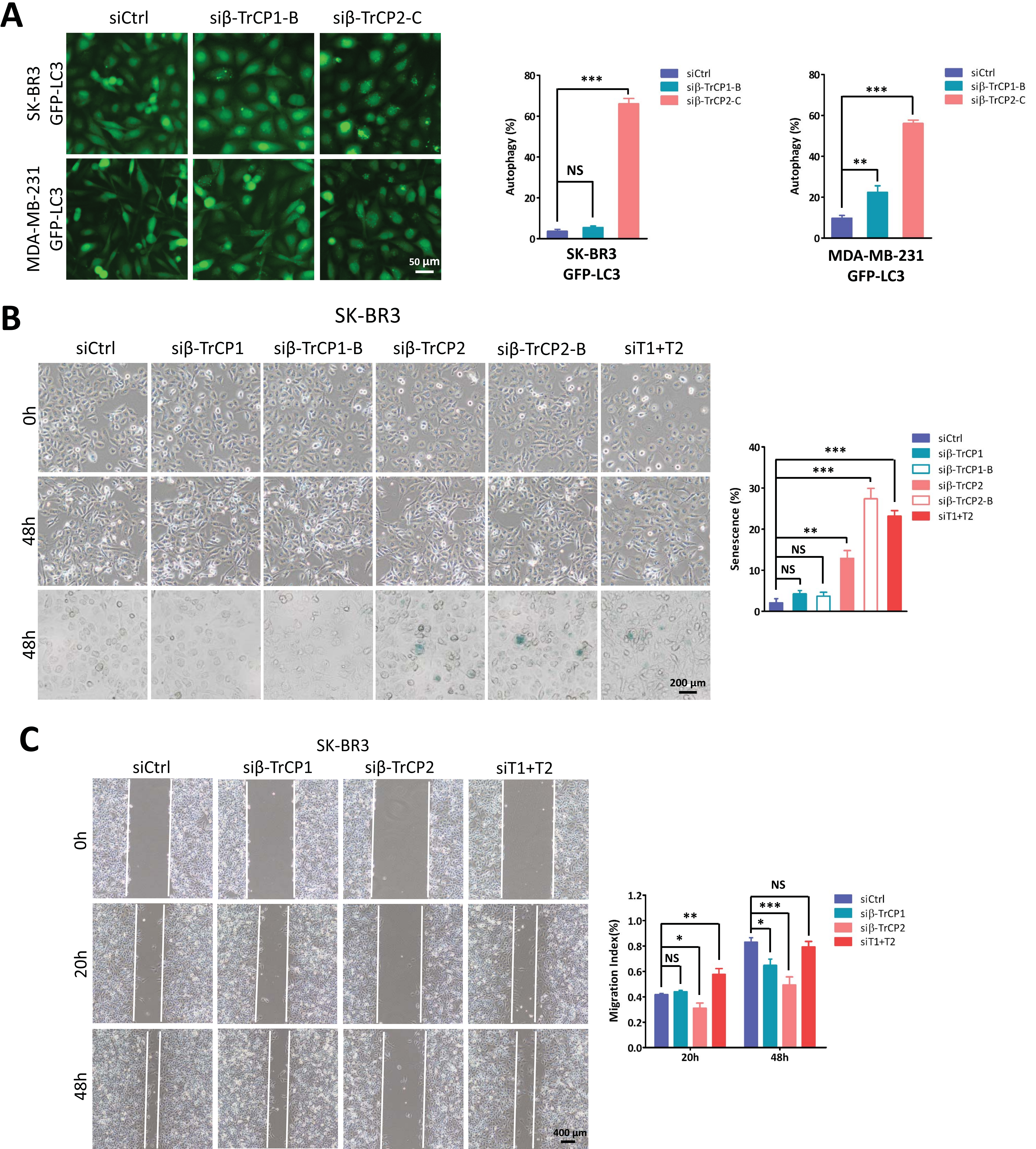

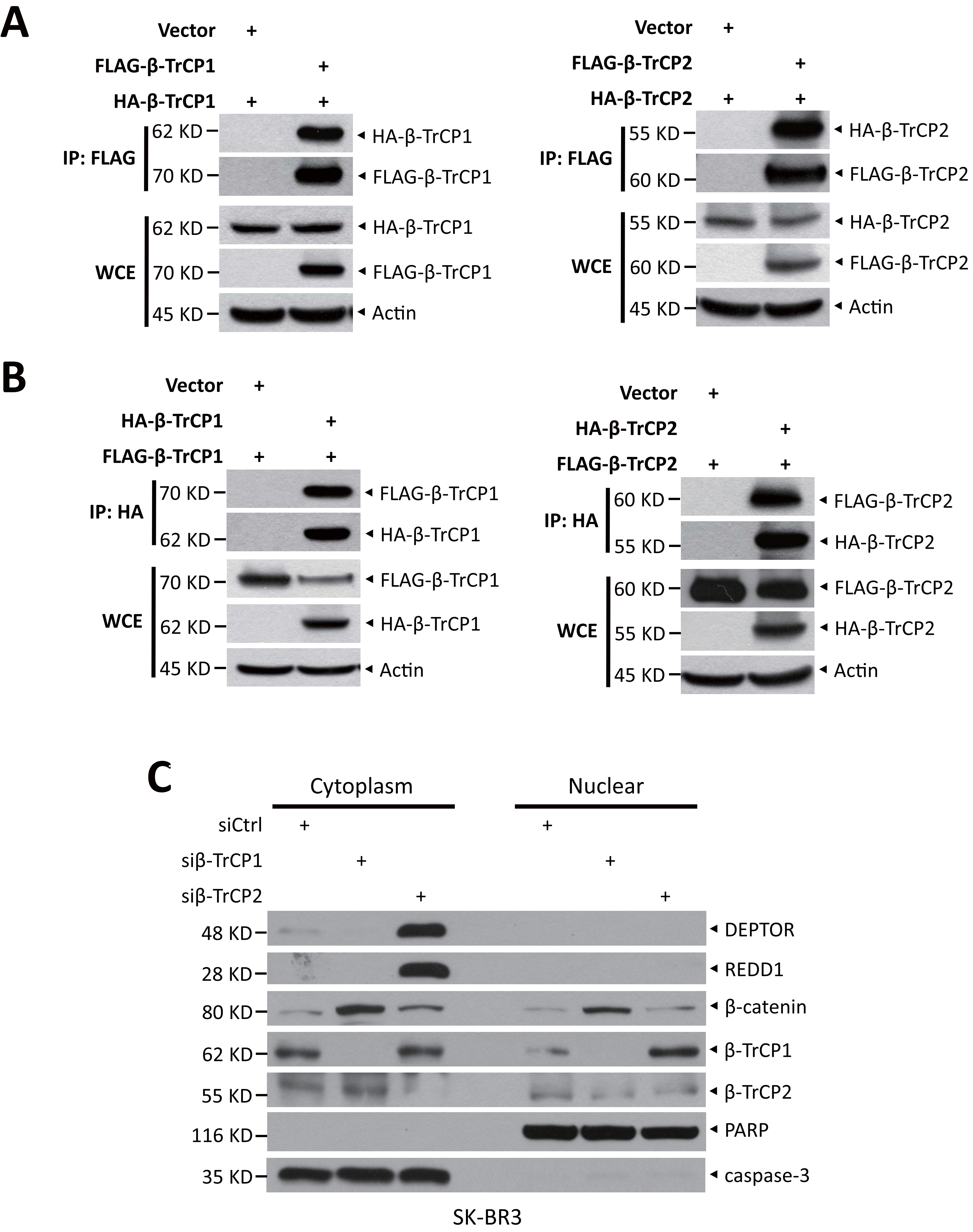
A
Vector +
FLAG-β-TrCP1 +
HA-β-TrCP1 + +
IP: FLAG
62 KD
70 KD
HA-β-TrCP1
FLAG-β-TrCP1
WCE
62 KD
70 KD
45 KD
HA-β-TrCP1
FLAG-β-TrCP1
Actin
Vector +
FLAG-β-TrCP2 +
HA-β-TrCP2 + +
IP: FLAG
55 KD
60 KD
HA-β-TrCP2
FLAG-β-TrCP2
WCE
55 KD
60 KD
45 KD
HA-β-TrCP2
FLAG-β-TrCP2
Actin
B
Vector +
HA-β-TrCP1 +
FLAG-β-TrCP1 + +
IP: HA
70 KD
62 KD
FLAG-β-TrCP1
HA-β-TrCP1
WCE
70 KD
62 KD
45 KD
FLAG-β-TrCP1
HA-β-TrCP1
Actin
Vector +
HA-β-TrCP2 +
FLAG-β-TrCP2 + +
IP: HA
60 KD
55 KD
FLAG-β-TrCP2
HA-β-TrCP2
WCE
60 KD
55 KD
45 KD
FLAG-β-TrCP2
HA-β-TrCP2
Actin
C
Cytoplasm
Nuclear
siCtrl + +
siβ-TrCP1 + +
siβ-TrCP2 + +
48 KD DEPTOR
28 KD REDD1
80 KD β-catenin
62 KD β-TrCP1
55 KD β-TrCP2
116 KD PARP
35 KD caspase-3
SK-BR3
